# Supplementary material for: A Critical View of the Application of the APEX Software (Aqueous Photochemistry of Environmentally-Occurring Xenobiotics) to Predict Photoreaction Kinetics in Surface Freshwaters
Source: Molecules. 2019 Dec 18;25(1):9. doi: 10.3390/molecules25010009 (PMC7017383; doi:10.3390/molecules25010009)
Supplement: Supplementary file 1 [file molecules-25-00009-s001.zip › 3.2.4_gcc-4.4.0.zip!/3.2.4_gcc-4.4.0/3.2.4_gcc-4.4.0/doc/octave/pdf/Octave-FAQ.pdf]

Octave FAQ

Frequently asked questions about Octave
September 2008

John W. Eaton and David Bateman

This is a list of frequently asked questions (FAQ) for Octave users.

We are always looking for new questions (*with* answers), better answers, or both. Please send suggestions to bug@octave.org. If you have general questions about Octave, or need help for something that is not covered by the Octave manual or the FAQ, please use the help@octave.org mailing list.

This FAQ is intended to supplement, not replace, the Octave manual. Before posting a question to the help@octave.org mailing list, you should first check to see if the topic is covered in the manual.

1 What is Octave?

Octave is a high-level interactive language, primarily intended for numerical computations that is mostly compatible with Matlab.[1]

Octave can do arithmetic for real, complex or integer-valued scalars and matrices, solve sets of nonlinear algebraic equations, integrate functions over finite and infinite intervals, and integrate systems of ordinary differential and differential-algebraic equations.

Octave uses the GNU readline library to handle reading and editing input. By default, the line editing commands are similar to the cursor movement commands used by GNU Emacs, and a vi-style line editing interface is also available. At the end of each session, the command history is saved, so that commands entered during previous sessions are not lost.

The Octave distribution includes a 590+ page Texinfo manual. Access to the complete text of the manual is available via the help command at the Octave prompt.

1.1 Who develops Octave?

Discussions about writing the software that would eventually become Octave started in about 1988 with James B. Rawlings and John W. Eaton at the University of Texas. John W. Eaton was the original author of Octave, starting full-time development in February 1992. He is still the primary maintainer. The community of users/developers has in addition contributed some code and fuels the discussion on the mailing lists help@octave.org (user forum), bug@octave.org (bug reports), maintainers@octave.org (development issues), and octave-dev@lists.sourceforge.net (all things related to the Octave Forge repository of user-contributed functions).

1.2 Why GNU Octave?

The GNU Project was launched in 1984 to develop a complete Unix-like operating system which is free software: the GNU system.

GNU is a recursive acronym for “GNU’s Not Unix”; it is pronounced guh-noo, approximately like canoe.

The Free Software Foundation (FSF) is the principal organizational sponsor of the GNU Project.

[1] Matlab is a registered trademark of The MathWorks, Inc.

Octave became GNU Octave in 1997 (beginning with version 2.0.6). This meant agreeing to consider Octave a part of the GNU Project and support the efforts of the FSF. However, Octave is not and has never been developed by the FSF.

For more information about the GNU project, see `www.gnu.org`.

1.3 What version should I use?

In general, you will find the latest version on `http://www.octave.org/download.html`. It is recommended to use the "testing" version of octave for general use, and the "development" version if you want the latest features.

A list of user-visible changes since the last release is available in the file '`NEWS`'. The file '`ChangeLog`' in the source distribution contains a more detailed record of changes made since the last release.

1.4 On what platforms does Octave run?

Octave runs on various Unices—at least Linux and Solaris, Mac OS X, Windows and anything you can compile it on. Binary distributions exist at least for Debian, Suse, Fedora and RedHat Linuxes (Intel and AMD CPUs, at least), for Mac Os X and Windows' 98, 2000 and XP.

Two and three dimensional plotting is fully supported using gnuplot.

The underlying numerical solvers are currently standard Fortran ones like Lapack, Linpack, Odepack, the Blas, etc., packaged in a library of C++ classes. If possible, the Fortran subroutines are compiled with the system's Fortran compiler, and called directly from the C++ functions. If that's not possible, you can still compile Octave if you have the free Fortran to C translator f2c.

Octave is also free software; you can redistribute it and/or modify it under the terms of the GNU General Public License as published by the Free Software Foundation.

2 Licensing Issues

2.1 If I write code using Octave do I have to release it under the GPL?

The answer depends on precisely how the code is written and how it works.

Code written entirely in the scripting language of Octave (interpreted code in .m files) may be released under the terms of whatever license you choose.

Code written using Octave's native plug-in interface (also known as a .oct file) necessarily links with Octave internals and is considered a derivative work of Octave and therefore must be released under terms that are compatible with the GPL.

Code written using Octave's implementation of the Matlab MEX interface may be released under the terms of whatever license you choose, provided that the following conditions are met:

1. The plugin should not use any bindings that are specific to Octave. In other words, the MEX file must use the MEX interface only, and not also call on other Octave internals. It should be possible in principle to use the MEX file with other programs that implement the MEX interface (e.g., Matlab).
2. The MEX file should not be distributed together with Octave in such a way that they effectively create a single work. For example, you should not distribute the MEX file and Octave together in a single package such that Octave automatically loads and runs the MEX file when it starts up. There are other possible ways that you might effectively create a single work; this is just one example.

A program that embeds the Octave interpreter (e.g., by calling the "octave_main" function), or that calls functions from Octave's libraries (e.g., liboctinterp, liboctave, or libcruft) is considered a derivative work of Octave and therefore must be released under terms that are compatible with the GPL.

2.2 Since the MEX interface allows plugins to be distributed under terms that are incompatible with the GPL, does this mean that you are encouraging people to to write non-free software for Octave?

No. The original reason for implementing the MEX interface for Octave was to allow Octave to run free software that uses MEX files (the particular goal was to run SundialsTB in Octave). The intent was to liberate that software from Matlab and increase the amount of free software available to Octave users, not to enable people to write proprietary code for Octave. For the good of the community, we strongly encourage users of Octave to release the code they write for Octave under terms that are compatible with the GPL.

2.3 I wrote a program that links with Octave libraries and I don't want to release it under the terms of the GPL. Will you change the license of the Octave libraries for me?

No. Instead of asking us to change the licensing terms for Octave, we recommend that you release your program under terms that are compatible with the GPL so that the free software community can benefit from your work the same as you have benefitted from the work of all the people who have contributed to Octave.

3 How can I cite Octave?

Pointing to `http://www.octave.org` is good, because that gives people a direct way to find out more. If citation of a URL is not allowed by a publisher, or if you also want to point to a traditional reference, then you can cite the Octave manual:

```
@BOOK{eaton:2008,
  author =     "John W. Eaton and David Bateman and Sren Hauberg",
  title =      "GNU Octave Manual Version 3",
  publisher =  "Network Theory Limited",
  year =       "2008",
  isbn =       "0-9546120-6-X"
}
```

4 What's new in version series 3.0.N and 3.1.N of Octave

The 3.0.N series has enough new features to justify a major version number change. The 3.0.N series brings

- integer types
- fixed point arithmetic
- sparse matrices
- Linear programming code based on GLPK
- 64-bit compilation support
- gzipped files and stream and consequently support of matlab v7 files
- better support for both msvc and mingw
- a fully compatible MEX interface
- many many other minor features and compatibility changes

Here are some features that have been around since 2.1.N

- NDarrays
- cells

The 3.1.N series is the current development release and will become a 3.2.N release in the future. This series brings the new features

- OpenGL backend

  An experimental OpenGL graphics backend to replace the gnuplot
- Object Orient Programming
- Block comments
- imwrite and imread

  The functions are based on the GraphicsMagick library.
- Lazy transpose

  Special treatment in the parser of things like "a' * b", where the transpose is never explicitly formed but a flag is rather passed to the underlying LAPACK code.
- Single precision type
- Improved array indexing The underlying code used for indexing of arrays has been completely rewritten and so the indexing of arrays is now significantly faster.

5 What features are unique to Octave?

This section refers to Matlab R2008b and Octave 2.1.51.

5.1 Functions defined on the command-line

Functions can be defined by entering code on the command line, a feature not supported by the other leading brand. For example, you may type:

```
octave:1> function s = hello_string (to_who)
> ## Say hello
> if nargin<1, to_who = "World"; end
> s = ["Hello ",\
>      to_who];
> endfunction
octave:2> hello_string ("Moon")
ans = Hello Moon
```

5.2 Comments with

The pound character, '`#`', may be used to start comments, in addition to '`%`'. See the previous example. The major advantage of this is that as '`#`' is also a comment character for unix script files, any file that starts with a string like '`#! /usr/bin/octave -q`' will be treated as an octave script and be executed by octave.

5.3 Strings delimitted by double quotes "

The double quote, '`"`', may be used to delimit strings, in addition to the single quote '`'`'. See the previous example. Also, double-quoted strings include backslash interpretation (like C++, C, and Perl) while single quoted are uninterpreted (like Matlab and Perl).

5.4 Line continuation by backslash

Lines can be continued with a backslash, '`\`', in addition to three points '`...`'. See the previous example.

5.5 Informative block closing

You may close `function`, `for`, `while`, `if`, . . . blocks with `endfunction`, `endfor`, `endwhile`, . . . keywords in addition to using `end`. As with Matlab, the `end` (or `endfunction`) keyword that marks the end of a function defined in a '`.m`' file is optional.

5.6 Coherent syntax

Indexing other things than variables is possible, as in:

```
octave:1> [3 1 4 1 5 9](3)
ans = 4
octave:2> cos([0 pi pi/4 7])(3)
ans = 0.70711
```

5.7 Exclamation mark as not operator

The exclamation mark '!' (aka "Bang!") is a negation operator, just like the tilde '~':

```
octave:1> if ! strcmp (program_name, "octave"),
>   "It's an error"
> else
>   "It works!"
> end
ans = It works!
```

5.8 Increment and decrement operators

If you like the '++', '+=' etc operators, rejoice! Octave includes the C-like increment and decrement operators '++' and '--' in both their prefix and postfix forms, in addition to '+=', '-=', '*=', '/=', '^=', '.*=', './=', and '.^='.

For example, to pre-increment the variable *x*, you would write `++x`. This would add one to *x* and then return the new value of *x* as the result of the expression. It is exactly the same as the expression `x = x + 1`.

To post-increment a variable *x*, you would write `x++`. This adds one to the variable *x*, but returns the value that *x* had prior to incrementing it. For example, if *x* is equal to 2, the result of the expression `x++` is 2, and the new value of *x* is 3.

For matrix and vector arguments, the increment and decrement operators work on each element of the operand.

5.9 Unwind-protect

Octave supports a limited form of exception handling modelled after the unwind-protect form of Lisp. The general form of an `unwind_protect` block looks like this:

```
unwind_protect
  body
unwind_protect_cleanup
  cleanup
end_unwind_protect
```

Where *body* and *cleanup* are both optional and may contain any Octave expressions or commands. The statements in *cleanup* are guaranteed to be executed regardless of how control exits *body*.

The `unwind_protect` statement is often used to reliably restore the values of global variables that need to be temporarily changed.

Matlab can be made to do something similar with their `OnCleanUp` function that was introduced in 2008a.

5.10 Built-in ODE and DAE solvers

Octave includes LSODE and DASSL for solving systems of stiff ordinary differential and differential-algebraic equations. These functions are built in to the interpreter.

6 What documentation exists for Octave?

6.1 What documentation exists for Octave?

The Octave distribution includes a 590+ page manual that is also distributed under the terms of the GNU GPL. It is available on the web at http://www.octave.org/docs.html and you will also find there instructions on how to order a paper version.

The complete text of the Octave manual is also available using the GNU Info system via the GNU Emacs, info, or xinfo programs, or by using the '`help -i`' command to start the GNU info browser directly from the Octave prompt.

If you have problems using this documentation, or find that some topic is not adequately explained, indexed, or cross-referenced, please send a bug report to bug@octave.org.

6.2 Getting additional help

If you can't find an answer to your question, the help@octave.org mailing list is available for questions related to using, installing, and porting Octave that are not adequately answered by the Octave manual or by this document.

6.3 User community

To subscribe to the list, go to www.octave.org/archive.html and follow the link to the subscription page for the list.

Please do not send requests to be added or removed from the mailing list, or other administrative trivia to the list itself.

An archive of old postings to the help-octave mailing list is maintained on http://www.octave.org/archive.html.

You will also find some user advice and code spread over the web. Good starting points are the Octave Wiki http://wiki.octave.org and Octave-Forge http://octave.sourceforge.net

6.4 I think I have found a bug in Octave.

"I think I have found a bug in Octave, but I'm not sure. How do I know, and who should I tell?"

First, see the section on bugs and bug reports in the Octave manual. When you report a bug, make sure to describe the type of computer you are using, the version of the operating system it is running, and the version of Octave that you are using. Also provide enough code so that the Octave maintainers can duplicate your bug.

If you have Octave working at all, the easiest way to do this is to use the Octave function `bug_report`. When you execute this function, Octave will prompt you for a subject and then invoke the editor on a file that already contains all the configuration information. When you exit the editor, Octave will mail the bug report for you (in a unix-like operating system).

If for some reason you cannot use Octave's `bug_report` function, mail your bug report to bug@octave.org. Your message needs to include enough information to allow the maintainers of Octave to fix the bug. Please read the section on bugs and bug reports in the Octave manual for a list of things that should be included in every bug report.

7 Getting Octave

7.1 Source code

Source code is available on the Octave development site, where you are sure to get the latest version.

- http://www.octave.org/download.html
- ftp://ftp.octave.org/pub/octave/

Since Octave is distrubted under the terms of the GPL, you can get Octave from a friend who has a copy, by anonymous FTP, or by ordering a tape or CD-ROM from the Free Software Foundation (FSF).

7.2 Pre-compiled binary packages

The Octave project does not distribute binary packages, but other projects do. For an up-to-date listing of packagers, see:

- http://www.octave.org/download.html
- http://wiki.octave.org/wiki.pl?CategoryInstall

As of today, Octave binaries are available at least on Debian, RedHat, Suse and Fedora Linuxes, Mac OS X, Windows' 98, 2000 and XP.

7.3 How do I get a copy of Octave for (some other platform)?

Octave currently runs on Unix-like systems, Mac OS X, and Windows. It should be possible to make Octave work on other systems as well. If you are interested in porting Octave to other systems, please contact bug@octave.org.

8 Installation Issues and Problems

Octave 3.2 require approximately 800MB of disk storage to unpack and compile from source (considerably less if you don't compile with debugging symbols). Once installed, Octave requires approximately 200MB of disk space (again, considerably less if you don't compile with debugging symbols).

8.1 What else do I need?

To compile Octave, you will need a recent version of GNU Make. You will also need GCC 3.3 or later, although GCC 4.1 or later is recommended.

You must have GNU Make to compile octave. Octave's Makefiles use features of GNU Make that are not present in other versions of make. GNU Make is very portable and easy to install.

8.2 Can I compile Octave with another C++ compiler?

Yes, but development is done primarily with GCC, so you may hit some incompatibilities. Octave is intended to be portable to any standard conforming compiler. If you have difficulties that you think are bugs, please report them to the bug@octave.org mailing list, or ask for help on the help@octave.org mailing list.

9 Common problems

This list is probably far too short. Feel free to suggest additional questions (preferably with answers!)

- Octave takes a long time to find symbols.

  Octave uses the `genpath` function to recursively add directories to the list of directories searched for function files. Check the list of directories with the `path` command. If the path list is very long check your use of the `genpath` function.

- When plotting Octave occasionally gives me errors like '`gnuplot> 9 0.735604 line 26317: invalid command`'.

  There is a known bug in gnuplot 4.2 that can cause an off by one error while piping data to gnuplot. The relevant gnuplot bug report can be found at http://sourceforge.net/tracker/index.php?func=detail&aid=1716556&group_id=2055&atid=102055

  If you have obtained your copy of Octave from a distribution please file a bug report requesting that the fix reported in the above bug report be included.

- I cannot install a package. Octave complains about a missing `mkoctfile`.

  Most distributions split Octave into several packages. The script `mkoctfile` is then part of a separate package:

  - Debian/Ubuntu

    ```
    aptitude install octave3.0-headers
    ```

  - Fedora

    ```
    yum install octave-devel
    ```

10 How do I ...?

10.1 How do I set the number of displayed decimals?

```
octave:1> format long
octave:2> pi
pi = 3.14159265358979
octave:3> format short
octave:4> pi
pi = 3.1416
```

11 Porting programs from Matlab to Octave

People often ask

"I wrote some code for Matlab, and I want to get it running under Octave. Is there anything I should watch out for?"

or alternatively

"I wrote some code in Octave, and want to share it with Matlab users. Is there anything I should watch out for?"

which is not quite the same thing. There are still a number of differences between Octave and Matlab, however in general differences between the two are considered as bugs. Octave might consider that the bug is in Matlab and do nothing about it, but generally functionality is almost identical. If you find a difference between Octave behavior and Matlab, then you should send a description of this difference (with code illustrating the difference, if possible) to bug@octave.org.

Furthermore, Octave adds a few syntactical extensions to Matlab that might cause some issues when exchanging files between Matlab and Octave users. As both Octave and Matlab are under constant development the information in this section is subject to change at anytime.

You should also look at the page http://octave.sourceforge.net/packages.html and http://octave.sourceforge.net/doc/ that has a function reference that is up to date. You can use this function reference to see the number of octave function that are available and their Matlab compatibility.

The major differences between Octave 3.2.N and Matlab R2008a are:

- Nested Functions

  Octave doesn't yet have nested functions. That is

  ```
  function y = foo (x)
    y = bar(x)
    function y = bar (x)
      y = ...;
    end
  end
  ```

  There was discussion in Octave of having these even prior to Matlab, and the decision was made not to have these in Octave at the time for compatibility. The above written with sub-functions functions would be

```
function y = foo (x)
   y = bar(x)
end
function y = bar (x)
   y = ...;
end
```

Now that Matlab has recently introduced nested functions, Octave will probably have them soon as well. Until then nested functions in Octave are treated as sub-functions with the same scoping rules as sub-functions.

The authors of Octave consider the nested function scoping rules of Matlab to be more problems than they are worth as they introduce diffiult to find bugs as inadvertantly modifying a variable in a nested function that is also used in the parent is particularly easy.

- Differences in core syntax There a few core Matlab syntaxes that are not accepted by Octave, these being
  - Some limitations on the use of function handles. The major difference is related to nested function scoping rules (as above) and their use with function handles.
  - Some limitations of variable argument lists on the LHS of an expression, though the most common types are accepted.
  - Matlab classdef object oriented programming is not yet supportted, though work is underway and when development more on to Octave 3.3 this will be included in teh development tree.
- Differences in core functions A large number of the Matlab core functions (ie those that are in the core and not a toolbox) are implemented, and certainly all of the commonly used ones. There are a few functions that aren't implemented, for example `condest` or to do with specific missing Octave functionality (gui, dll, java, activex, dde, web, and serial functions). Some of the core functions have limitations that aren't in the Matlab version. For example the `sprandn` function can not force a particular condition number for the matrix like Matlab can.
- Just-In-Time compiler Matlab includes a "Just-In-Time" compiler. This compiler allows the acceleration of for-loops in Matlab to almost native performance with certain restrictions. The JIT must know the return type of all functions called in the loops and so you can't include user functions in the loop of JIT optimized loops. Octave doesn't have a JIT and so to some might seem slower than Matlab. For this reason you must vectorize your code as much as possible. The MathWorks themselves have a good document discussing vectorization at `http://www.mathworks.com/support/tech-notes/1100/1109.html`.
- Compiler On a related point, there is no Octave compiler, and so you can't convert your Octave code into a binary for additional speed or distribution. There is an example of how to do this at `http://www.stud.tu-ilmenau.de/~rueckn/`, but this is a very early example code and would need lots of work to complete it.
- Graphic Handles Up to Octave 2.9.9 there was no support for graphic handles in Octave itself. In the 3.2.N versions of Octave the support for graphics handles is converging towards full compatibility. The `patch` function is currently limited to 2-D patches, due to an underlying limitation in gnuplot.

- GUI There are no Matlab compatible GUI functions. There are a number of bindings from Octave to Tcl/Tk, Vtk and zenity included in the Octave Forge project (http://octave.sourceforge.net) for example that can be used for a GUI, but these are not Matlab compatible. Work on a matlab compatible GUI is in an alpha stage in the JHandles package (http://octave.sourceforge.net/jhandles/index.html). This might be an issue if you intend to exchange Octave code with Matlab users.
- Simulink Octave itself includes no Simulink support. Typically the simulink models lag research and are less flexible, so shouldn't really be used in a research environment. However, some Matlab users that try to use Octave complain about this lack. There is a similar package to simulink for the Octave and R projects available at http://www.scicraft.org/
- Mex-Files Octave includes an API to the matlab MEX interface. However, as MEX is an API to the internals of Matlab and the internals of Octave differ from Matlab, there is necessarily a manipulation of the data to convert from a MEX interface to the Octave equivalent. This is notable for all complex matrices, where Matlab stores complex arrays as real and imaginary parts, whereas Octave respects the C99/C++ standards of co-locating the real/imag parts in memory. Also due to the way Matlab allows access to the arrays passed through a pointer, the MEX interface might require copies of arrays (even non complex ones).
- Block comments Block comments denoted by "%{" and "%}" markers are supported by Octave with some limitations. The major limitation is that block comments are not supported within [] or `{}`.
- Mat-File format There are some differences in the mat v5 file format accepted by Octave. Matlab recently introduced the "-V7.3" save option which is an HDF5 format which is particularly useful for 64-bit platforms where the standard matlab format can not correctly save variables.. Octave accepts HDF5 files, but is not yet compatible with the "-v7.3" versions produced by Matlab.

  Although Octave can load inline abd function handles saved by Matlab, it can not yet save them.

  Finally, Some multi-byte unicode characters aren't yet treated in mat-files.
- Profiler Octave doesn't have a profiler. Though there is a patch for a flat profiler, that might become a real profiler sometime in the future. see the thread

  http://www.cae.wisc.edu/pipermail/octave-maintainers/2007-January/001685.html

  for more details
- Toolboxes Octave is a community project and so the toolboxes that exist are donated by those interested in them through the Octave Forge website (http://octave.sourceforge.net). These might be lacking in certain functionality relative to the Matlab toolboxes, and might not exactly duplicate the matlab functionality or interface.
- Short-circuit & and | operators The `&` and `|` operators in Matlab short-circuit when included in an if statemant and not otherwise. In Octave only the `&&` and `||` short circuit. Note that this means that

  ```
  if (a | b)
    ...
  end
  ```

and

```
t = a | b;
if t
  ...
end
```

are different in Matlab. This is really a Matlab bug, but there is too much code out there that relies on this behavior to change it. Prefer the || and && operators in if statements if possible.

Note that the difference is also significant when either argument is a function with side effects or if the first argument is a scalar and the second argument is an empty matrix. For example, note the difference between

```
t = 1 | [];          ## results in [], so...
if (t) 1, end        ## in if ([]), this is false.
```

and

```
if (1 | []) 1, end   ## short circuits so condition is true.
```

Another case that is documented in the Matlab manuals is that

```
t = [1, 1] | [1, 2, 3];          ## error
if ([1, 1] | [1, 2, 3]) 1, end   ## OK
```

Also Matlab requires the operands of && and || to be scalar values but Octave does not (it just applies the rule that for an operand to be considered true, every element of the object must be nonzero or logically true).

Finally, note the inconsistence of thinking of the condition of an if statement as being equivalent to `all(X(:))` when X is a matrix. This is true for all cases EXCEPT empty matrices:

```
if ([0, 1]) == if (all ([0, 1]))   ==>  i.e., condition is false.
if ([1, 1]) == if (all ([1, 1]))   ==>  i.e., condition is true.
```

However,

```
if ([]) != if (all ([]))
```

because `samp ([]) == 1` (because, despite the name, it is really returning true if none of the elements of the matrix are zero, and since there are no elements, well, none of them are zero). But, somewhere along the line, someone decided that if `([])` should be false. Mathworks probably thought it just looks wrong to have `[]` be true in this context even if you can use logical gymnastics to convince yourself that "all" the elements of a matrix that doesn't actually have any elements are nonzero. Octave however duplicates this behavior for if statements containing empty matrices.

- Solvers for singular, under- and over-determined matrices

  Matlab's solvers as used by the operators mldivide (\) and mrdivide (/), use a different approach than Octave's in the case of singular, under-, or over-determined matrices. In the case of a singular matrix, Matlab returns the result given by the LU decomposition, even though the underlying solver has flagged the result as erroneous. Octave has made the choice of falling back to a minimum norm solution of matrices that have been flagged as singular which arguably is a better result for these cases.

  In the case of under- or over-determined matrices, Octave continues to use a minimum norm solution, whereas Matlab uses an approach that is equivalent to

```
function x = mldivide (A, b)
  [Q, R, E] = qr(A);
  x = [A \ b, E(:, 1:m) * (R(:, 1:m) \ (Q' * b))]
end
```

While this approach is certainly faster and uses less memory than Octave's minimum norm approach, this approach seems to be inferior in other ways.

A numerical question arises: how big can the null space component become, relative to the minimum-norm solution? Can it be nicely bounded, or can it be arbitrarily big? Consider this example:

```
m = 10;
n = 10000;
A = ones(m, n) + 1e-6 * randn(m,n);
b = ones(m, 1) + 1e-6 * randn(m,1);
norm(A \ b)
```

while Octave's minimum-norm values are around 3e-2, Matlab's results are 50-times larger. For another issue, try this code:

```
m = 5;
n = 100;
j = floor(m * rand(1, n)) + 1;
b = ones(m, 1);
A = zeros(m, n);
A(sub2ind(size(A),j,1:n)) = 1;
x = A \ b;
[dummy,p] = sort(rand(1,n));
y = A(:,p)\b;
norm(x(p)-y)
```

It shows that unlike in Octave, mldivide in Matlab is not invariant with respect to column permutations. If there are multiple columns of the same norm, permuting columns of the matrix gets you different result than permuting the solution vector. This will surprise many users.

Since the mldivide (\) and mrdivide (/) operators are often part of a more complex expression, where there is no room to react to warnings or flags, it should prefer intelligence (robustness) to speed, and so the Octave developers are firmly of the opinion that Octave's approach for singular, under- and over-determined matrices is a better choice that Matlab's

- Octave extensions The extensions in Octave over Matlab syntax are very useful, but might cause issues when sharing with Matlab users. A list of the major extensions that should be avoided to be compatible with Matlab are
  - Comments in octave can be marked with '`#`'. This allows POSIX systems to have the first line as '`#! octave -q`' and mark the script itself executable. Matlab doesn't have this feature due to the absence of comments starting with '`#`'".
  - Code blocks like if, for, while, etc can be terminated with block specific terminations like "endif". Matlab doesn't have this and all blocks must be terminated with "end"

- Octave has a lisp like unwind_protect block that allows blocks of code that terminate in an error to ensure that the variables that are touched are restored. You can do something similar with `try/catch` combined with '`rethrow (lasterror ())`' in Matlab, however rethrow and lasterror are only available in Octave 2.9.10 and later.

  Note that using `try/catch` combined with '`rethrow (lasterror ())`' can not guarantee that global variables will be correctly reset, as it won't catch user interrupts with Ctrl-C. For example

  ```
  global a
  a = 1;
  try
    _a = a;
    a = 2
    while true
    end
  catch
    fprintf ('caught interrupt\n');
    a = _a;
    rethrow (lasterror());
  end
  ```

  compared to

  ```
  global a
  a = 1;
  unwind_protect
    _a = a;
    a = 2
    while true
    end
  unwind_protect_cleanup
    fprintf ('caught interrupt\n');
    a = _a;
  end
  ```

  Typing Ctrl-C in the first case returns the user directly to the prompt, and the variable "a" is not reset to the saved value. In the second case the variable "a" is reset correctly. Therefore Matlab gives no save way of temporarily changing global variables.

- Indexing can be applied to all objects in Octave and not just variable. Therefore `sin(x)(1:10);` for example is perfectly valid in Octave but not Matlab. To do the same in Matlab you must do `y = sin(x); y = y([1:10]);`

- Octave has the operators "++", "–", "-=", "+=", "*=", etc. As Matlab doesn't, if you are sharing code these should be avoided.

- Character strings in Octave can be denoted with double or single quotes. There is a subtle difference between the two in that escaped characters like `\n` (newline), `\t` (tab), etc are interpreted in double quoted strings but not single quoted strings. This difference is important on Windows platforms where the "\" character is used

in path names, and so single quoted strings should be used in paths. MATLAB doesn't have double quoted strings and so they should be avoided if the code will be transfered to a MATLAB user.

Appendix A Concept Index

A

Additional help ... 7

B

Binaries ... 8
Bug in Octave, newly found ... 7

C

Compatibility with MATLAB ... 10

D

DASSL ... 6
Decrement operators ... 6
DJGPP ... 8

E

EMX ... 8

F

Flex ... 9
FSF [Free Software Foundation] ... 2

G

GNU [GNU's not unix] ... 2
GNU Bison ... 9
GNU g++ ... 9
GNU gcc ... 9
GNU Make ... 9

H

How do I ... ? ... 10

I

Increment operators ... 6

L

libg++ ... 9
LSODE ... 6

M

Mailing lists, bug-octave ... 7
Mailing lists, help-octave ... 7
Manual, for Octave ... 7
MATLAB compatibility ... 10
MS-DOS support ... 8

O

Octave bug report ... 7
Octave, building ... 8
Octave, documentation ... 7
Operators, decrement ... 6
Operators, increment ... 6
OS/2 support ... 8

P

Pre-compiled binary packages ... 8

S

Source code ... 8

T

Tips and tricks ... 10

U

Unwind-protect ... 6

V

VAX ... 8
VMS support ... 8

W

Windows support ... 8

Table of Contents

1 What is Octave? 1

1.1 Who develops Octave? 1
1.2 Why GNU Octave? 1
1.3 What version should I use? 2
1.4 On what platforms does Octave run? 2

2 Licensing Issues 2

2.1 If I write code using Octave do I have to release it under the GPL? 2
2.2 Since the MEX interface allows plugins to be distributed under terms that are incompatible with the GPL, does this mean that you are encouraging people to to write non-free software for Octave? 3
2.3 I wrote a program that links with Octave libraries and I don't want to release it under the terms of the GPL. Will you change the license of the Octave libraries for me? 3

3 How can I cite Octave? 3

4 What's new in version series 3.0.N and 3.1.N of Octave 4

5 What features are unique to Octave? 5

5.1 Functions defined on the command-line 5
5.2 Comments with # 5
5.3 Strings delimitted by double quotes " 5
5.4 Line continuation by backslash 5
5.5 Informative block closing 5
5.6 Coherent syntax 5
5.7 Exclamation mark as not operator 6
5.8 Increment and decrement operators 6
5.9 Unwind-protect 6
5.10 Built-in ODE and DAE solvers 6

6 What documentation exists for Octave? 7

6.1 What documentation exists for Octave? 7
6.2 Getting additional help 7
6.3 User community 7
6.4 I think I have found a bug in Octave. 7

7 Getting Octave **8**

7.1 Source code 8
7.2 Pre-compiled binary packages 8
7.3 How do I get a copy of Octave for (some other platform)? 8

8 Installation Issues and Problems **8**

8.1 What else do I need? 9
8.2 Can I compile Octave with another C++ compiler? 9

9 Common problems **9**

10 How do I ...? **10**

10.1 How do I set the number of displayed decimals? 10

11 Porting programs from MATLAB to Octave **10**

Appendix A Concept Index **16**
